# Does having various types of fear related to COVID-19 disrupt individuals’ daily life?: Findings from a nationwide survey in Korea

**DOI:** 10.4178/epih.e2022004

**Published:** 2022-01-03

**Authors:** Woorim Kim, Yeong Jun Ju, Soon Young Lee

**Affiliations:** 1 Division of Cancer Control & Policy, National Cancer Control Institute, National Cancer Center, Goyang, Korea; 2 Department of Preventive Medicine and Public Health, Ajou University School of Medicine, Suwon, Korea

**Keywords:** Coronavirus, Fear, Activities of daily living, Income

## Abstract

**OBJECTIVES:**

Unexpected changes in daily routines caused by the coronavirus disease 2019 (COVID-19) pandemic may affect psychological health. This study investigated the association between various types of COVID-19-related fear and the subjective level of disturbance in daily activities experienced by individuals.

**METHODS:**

This cross-sectional study used the Korea Community Health Survey conducted from August through November 2020. COVID-19-related fear included fear of infection, death, public criticism, family members getting infected, and economic loss. The subjective level of disruption in daily activities was measured using a 0-100 numeric rating scale developed by the Korea Disease Control and Prevention Agency. Multivariable linear regression was used to analyze the associations between the independent and dependent variables. A subgroup analysis was performed based on income level.

**RESULTS:**

Participants who reported fear of infection (β=-3.37, p<0.001), death (β=-0.33, p=0.030), public criticism (β=-1.63, p<0.001), a family member getting infected (β=-1.03, p<0.001), and economic loss (β=-3.52, p<0.001) experienced more disturbances in daily activities. The magnitude of this association was most significant in the lowest-income group.

**CONCLUSIONS:**

Individuals reporting COVID-19-related fear experienced higher levels of subjective disruption in daily activities.

## INTRODUCTION

In March 2020, the World Health Organization declared the coronavirus disease 2019 (COVID-19), which has infected many people since its abrupt emergence in December 2019, to be a global pandemic [1]. In an effort to flatten the rapid escalation in the number of confirmed cases and deaths, many countries, including Korea, implemented strict policies on social distancing, conducted public awareness and personal hygiene campaigns, and introduced swift case testing and isolation protocols [2]. Similar governmental responses were taken worldwide, and countries achieved varying degrees of success in managing the pandemic depending on their preparedness and responsiveness [3].

While social distancing and isolation strategies have been instrumental in containing the rate of transmission, they inevitably led to abrupt changes in the daily lives of numerous individuals [4]. The widespread social effects of the pandemic created confusion and disruptions in daily activities among the general population [5]. For instance, social distancing and isolation policies reduced interpersonal interactions, introduced novel work patterns, caused the suspension of schools, and endangered basic livelihoods [6]. Since regular routines are known to normalize the general structure of daily living, such alterations and disruptions can interfere with an individual’s well-being and mental health [6,7]. Unsurprisingly, studies have shown that the unprecedented changes caused by the pandemic impacted the psychological aspects and mental health of many people [8]. However, individuals may experience different levels of disruption due to differences in resilience capacity, which refers to the process of managing everyday life stressors [9]. Hence, there is a need to address the level of disruption in daily life reported during the outbreak and identify and manage particularly vulnerable groups or individuals.

Fear, a natural response activated during stressful times such as a pandemic or an outbreak, can be associated with the extent of changes in daily life behaviors [5,10]. COVID-19 can cause various types of fear, including those associated with family well-being, infection and hygiene, or the need to conform to the rules to avoid criticism [11]. COVID-19 related fear may have perceived benefits, such as better engagement in preventive hygiene behaviors and compliance to public health measures [12]. However, widespread, excessive, or chronic fear impacted by disruptions in daily routines may impair psychological well-being and quality of life [13]. Extreme fear has also been associated with depressive symptoms and perceived job security, suggesting that it is necessary to manage fear as the uncertainty of the pandemic continues [14].

This study aimed to examine the association between COVID-19 related fear and the subjective level of disruption in daily life experienced by the general population. The various types of fear, which included fear of infection, dying from infection, public criticism, a family member getting infected, and economic loss, were investigated separately and concurrently. We hypothesized that individuals with a higher level of fear would report a higher level of disruption in daily life. A subgroup analysis was conducted based on income level.

## MATERIALS AND METHODS

### Data and study population

This study used raw data from the 2020 Korea Community Health Survey (KCHS) conducted by the Korea Disease Control and Prevention Agency. The KCHS is a cross-sectional survey, with participants from multistage, stratified area probability samples of civilian, non-institutionalized Korean households categorized according to geographic area, age, and sex. The survey is conducted annually and collects data through in-person (one-on-one) interviews. Since the population sample is extracted from national survey data, it is considered representative of the Korean population [15]. This study included individuals aged 19 years and older. From an initial total of 229,269 potential participants, those with missing data were excluded, and a total of 207,239 participants were included in the present study.

#### Dependent variable

The dependent variable was the subjective level of disruption in daily life experienced by the study participants. This variable was measured using a 0-100 numeric rating scale developed by the Korea Disease Control and Prevention Agency. The dependent variable was measured by asking the study participants, “Assuming that a score of 100 indicates no change in your daily life before and after the outbreak of COVID-19, whereas a score of 0 implies a complete change (a complete stop in carrying out previous activities in daily life before COVID-19), what do you think is your current status?”

#### Independent variable

The independent variable was COVID-19-related fear, which included fear of infection, dying from infection, public criticism, a family member getting infected, and economic loss. Each item was measured in response to the following statements: “I fear that I will get infected with COVID-19,” “I fear that I might die if I get infected,” “I fear that I may be criticized if I get infected,” “I fear that my family members vulnerable to poor health may get infected,” and “I fear that the outbreak may cause economic loss to me or my family.” Each item was considered separately and concurrently (the sum of the number of COVID-19 related fears was expressed between 0 to 5) using different models.

#### Covariates

Various socio-demographic and socioeconomic variables were included as covariates. These were sex (male or female), age (19-29, 30-39, 40-49, 50-59, 60-69, or ≥ 70), education (no diploma, elementary school, middle school, high school, or college or higher), income level (quartiles), job classification (professional or administrative work, office work, sales and service, agriculture and fishery, blue-collar work or simple labor, or unemployed), household composition (1-, 2-, or 3-generation household), area of residence (rural or urban), drinking at least once per month (no or yes), smoking (no or yes), depressive symptoms (no or yes), perceived stress (no or yes), and subjective health status (poor or fair). Depressive symptoms were measured using the Patient Health Questionnaire-9 (PHQ-9), with a score of 10 or above indicating depressive symptoms [16,17].

### Statistical analysis

We conducted the t-test and analysis of variance to examine the general characteristics of the participants; the mean and standard deviation of the dependent variable were calculated and compared between groups. The Cronbach alpha coefficient was used to confirm the internal consistency of the scale used to examine COVID-19-related fear in this study. Pearson correlation coefficients between each of the components included were also calculated to confirm the internal homogeneity of the scale measuring COVID-19-related fear. Multivariable linear regression analysis was also conducted to investigate the association between the subjective level of disruption in daily life and COVID-19-related fear. Each of the 5 types of fear was analyzed separately and concurrently (the sum of the number of fears experienced) in separate models while adjusting for all covariates. A subgroup analysis was conducted based on income level. The p-values were considered significant at p < 0.05. All analyses were performed using SAS version 9.4 (SAS Institute Inc., Cary, NC, USA).

### Ethics statement

The KCHS is open data where all personal information is fully anonymized before release. This study was covered under the review list pursuant to Article 2.2 of the Enforcement Rule of Bioethics and Safety Act in Korea, since the data were exempted from institutional review board review. This study was conducted in accordance with the ethical standards of the national research committee, the 1964 Helsinki Declaration, and its later amendments or comparable ethical standards.

## RESULTS

The general characteristics of the study participants are shown in Table 1. Of the 207,239 participants included, 71.3% feared COVID-19 infection, 45.9% feared dying from infection, 76.3% feared public criticism, 86.3% feared a family member getting infected, and 79.5% feared economic loss. The mean score of disruption in daily activities was 55.20± 23.06. Lower scores, which indicated a higher level of subjective changes in daily life, were found in individuals who reported fear. The internal consistency and internal homogeneity of the scale used to measure COVID-19-related fear in this study are shown in Table 2. Regarding internal consistency, the Cronbach alpha coefficient was 0.73. The Pearson correlation coefficients between each of the included components ranged between 0.31 to 0.48. These correlation values imply reasonable internal consistency and internal homogeneity of the scale utilized to measure COVID-19-related fear.

The results of the multivariable linear regression analysis of the association between COVID-19-related fear and the subjective level of disruption in daily life are presented in Tables 3 and 4. Compared to individuals who reported no fear, those with fears of COVID-19 infection (β= -3.37, p< 0.001), dying from infection (β= -0.33, p= 0.030), public criticism (β= -1.63, p< 0.001), a family member getting infected (β= -1.03, p< 0.001), and economic loss (β= -3.52, p< 0.001) had statistically significantly lower scores for disruptions in daily activities. When considering the different types of COVID-19-related fear concurrently, scores on the subjective level of disruption in daily activities decreased in a stepwise manner as the number of reported COVID-19-related fears increased (1 type of fear: β= -2.71, p< 0.001; 2 types of fear: β= -5.31, p< 0.001; 3 types of fear: β= -7.41, p< 0.001; 4 types of fear: β= -9.78, p< 0.001; 5 types of fear: β= -10.34, p< 0.001).

The results of the subgroup analysis performed based on income level are shown in Table 5. The general tendencies of the main findings shown in Table 4 were maintained regardless of income level, as scores tended to decline with the number of types of COVID-19-related fear that participants experienced. However, the magnitude of this decrease was most significant in the low-income group, followed by the middle-low, middle-high, and high-income groups.

## DISCUSSION

Our results revealed that fear related to the COVID-19 pandemic was associated with increased levels of subjective disruptions in daily activities. Compared to individuals without fear, those with fears of COVID-19 infection, dying from infection, public criticism, a family member getting infected, and economic loss reported higher levels of disruption in daily activities. Those who reported fear of COVID-19 infection and economic loss due to infection had particularly higher levels of disturbances. Considering the various types of fear concurrently, the level of disruption experienced tended to increase with the number of reported fears. Furthermore, this increased level of disruption was most strongly experienced in the lowest income group, followed by the low-middle, middle-high, and high-income groups.

Various types of fear have been commonly reported during the pandemic, including fears of infection, death due to infection, and public criticism [18,19]. Fear is a normal response to an emerging threat that allows individuals to promptly react to potential harms or threats [20]. Although fear can have a positive effect on the general population, such as better compliance with public health recommendations, excessive fear may exert a damaging impact by causing overreactions or impacting perceived health status [21]. Previous research has shown that COVID-19-related fear was also associated with life satisfaction, meaning in life, and hope [22]. The results of this study add evidence on this subject by suggesting a relationship between fear related to the pandemic and the subjective level of disruption experienced by the general population.

The positive correlation between fear related to the pandemic and disruptions in daily life may be partially explained by the fact that certain types of fear, such as the fear of infection or public criticism, can prompt individuals to adhere to a restricted lifestyle promoting infection control behaviors [23]. Social desirability, defined as the pressure applied to an individual to follow a norm, may induce people to comply with public health measures implemented by the government [24]. The abovementioned positive correlation is significant because studies have shown that disturbances in daily life can impact personal well-being and psychological status during an outbreak [25]. Studies have revealed that negative alterations in lifestyle habits can exert a psychological influence, such as increased symptoms of depression, anxiety, and stress, in the COVID-19 pandemic [26]. Moreover, deterioration of daily work activities can lead to decreased social interactions, which can increase psychological problems in many individuals [22]. The negative mental health impact of COVID-19 has been examined in different countries, and findings suggest the importance of monitoring and addressing fear and the level of daily life disruption reported by the general public during the pandemic.

The tendency for individuals who had COVID-19-related fear to report higher levels of disturbances in their daily lives was more pronounced in the low-income group. Low income was found to be associated with a higher risk of suffering from irregular daily rhythms, impacted by the weaker competitiveness of lower-income individuals in the employment market [27]. Furthermore, lower-income workers were reported to be exposed to a higher risk of infection since they were generally less able to work from home [28]. Unsurprisingly, economically marginalized populations carry a higher level of COVID-19 burden as they have less financial security, and losing income can lead to insecurity [29]. Hence, the relationship between fear related to the outbreak and the level of daily life disruptions experienced may have been particularly strong in this economic group. This implies that individuals from economically disadvantaged backgrounds may face a comparatively higher risk of daily life interruptions and related negative mental health effects. Low-income groups may be particularly vulnerable, as a bidirectional relationship has also been found between poverty and psychological health, and the pandemic is likely to aggravate the risk factors for poverty and expose individuals with lower incomes to a higher risk of mental disorders [30].

This study has some limitations. First, since the study design was cross-sectional, causal inferences based on the analysis should be made with caution. Second, the KCHS data were collected throughout the year, and the number of confirmed cases fluctuated. Hence, not all responses may have been recorded at identical times and situations, particularly because the Korean government also frequently adjusted its social distancing policy based on the number of cases. Third, responses on COVID-19-related fear were based solely on self-reports. However, various aspects of fear commonly reported during the outbreak were considered in the analysis. Fourth, due to the unprecedented pandemic situation, scales to evaluate the impact of COVID-19 were rapidly developed in many countries, tailored to the needs and characteristics of each country. The scale used to measure COVID-19-related fear and disruptions in daily activities due to the pandemic in this study was developed by the Korea Disease Control and Prevention Agency to investigate the impact of COVID-19 in Korea. The rapid development and utilization of these scales have led to inevitable limitations in testing their reliability and validity, particularly in studies targeting the general population. Although there were limitations in evaluating the validity and reliability of these measures at the researcher level, these scales are important and meaningful in that they can be used to investigate the impacts of COVID-19 on the general population. Hence, this study offers unique insights by revealing a positive relationship between fear caused by the pandemic and subjective levels of disturbance in daily living experienced by the general population.

In conclusion, individuals who reported fear related to COVID-19 experienced higher levels of subjective disturbance in daily activities during the outbreak. This increase was particularly higher among people who feared getting infected or had a fear of economic loss. Additionally, the magnitude of this association was stronger in lower-income groups. Since these disturbances experienced during a pandemic can have various psychological impacts, the findings of this study suggest that it is necessary to manage excessive fear and identify and monitor potentially vulnerable groups/individuals.

## Figures and Tables

**Table 1.** General characteristics of the study participants

Characteristics	Total	Disruption in daily activities	p-value	Characteristics	Total	Disruption in daily activities	p-value
Fear of infection			<0.001	Middle-high	51,237 (24.7)	53.85±22.20	
No	59,545 (28.7)	59.31±23.24		High	57,482 (27.7)	53.66±21.36	
Yes	147,694 (71.3)	53.54±22.77		Job classification			<0.001
Fear of dying from infection			<0.001	Professional or administrative work	21,166 (10.2)	53.19±21.38	
No	112,139 (54.1)	56.25±22.90		Office work	18,161 (8.8)	54.35±20.44	
Yes	95,100 (45.9)	53.97±23.18		Sales and service	26,228 (12.7)	51.88±22.43	
Fear of public criticism			<0.001	Agriculture and fishery	20,647 (9.9)	60.73±23.55	
No	49,031 (23.7)	58.31±23.52		Blue-collar work or simple labor	38,909 (18.8)	56.51±22.35	
Yes	158,208 (76.3)	54.23±22.83		Unemployed	82,128 (39.6)	54.96±24.12	
Fear of a family member getting infected			<0.001	Household composition			<0.001
No	28,365 (13.7)	59.46±23.17		One generation	97,490 (47.0)	57.01±23.81	
Yes	178,874 (86.3)	54.52±22.97		Two generations	95,295 (46.0)	53.42±22.18	
Fear of economic loss due to infection			<0.001	Three generations	14,454 (7.0)	54.73±22.62	
No	42,492 (20.5)	59.32±22.81		Area of residence			<0.001
Yes	164,747 (79.5)	54.14±23.00		Rural	91,589 (44.2)	58.03±23.69	
Sex			<0.001	Urban	115,650 (55.8)	52.96±22.29	
Male	93,996 (45.4)	56.61±22.73		Drinking at least once per month			<0.001
Female	113,243 (54.6)	54.03±23.26		No	114,396 (55.2)	56.04±23.64	
Age (yr)			<0.001	Yes	92,843 (44.8)	54.17±22.28	
19-29	23,065 (11.1)	52.95±21.60		Smoking			0.707
30-39	23,627 (11.4)	50.56±22.06		No	174,093 (84.0)	55.21±22.97	
40-49	33,186 (16.0)	52.93±21.46		Yes	33,146 (16.0)	55.16±23.52	
50-59	39,265 (19.0)	54.47±22.55		Depressive symptoms (PHQ-9 ≥10)			<0.001
60-69	40,586 (19.6)	55.53±23.73		No	201,396 (97.2)	55.35±22.91	
≥70	47,510 (22.9)	60.51±24.13		Yes	5,843 (2.8)	49.99±27.21	
Education			<0.001	Perceived stress			<0.001
No diploma	18,435 (8.9)	62.38±24.52		No	161,088 (77.7)	56.68±22.65	
Elementary school	30,297 (14.6)	58.40±24.22		Yes	46,151 (22.3)	50.05±23.71	
Middle school	22,663 (10.9)	55.89±23.76		Subjective health status			0.051
High school	70,193 (33.9)	53.87±22.70		Poor	108,200 (52.2)	55.11±23.33	
College or above	65,651 (31.7)	52.90±21.60		Fair	99,039 (47.8)	55.30±22.76	
Income level			<0.001	Total	207,239 (100)	55.20±23.06	
Low	51,478 (24.8)	58.46±24.96					
Low-middle	47,042 (22.8)	54.98±23.44					

Values are presented as number (%) or mean±standard deviation. PHQ-9, Patient Health Questionnaire-9.

**Table 2.** Item correlations of the scale used to measure coronavirus disease 2019 related fear

	C1	C2	C3	C4	C5
C1 Fear of infection (total)	1.00	-	-	-	-
Male	1.00	-	-	-	-
Female	1.00	-	-	-	-
C2 Fear of dying from infection (total)	0.48	1.00	-	-	-
Male	0.50	1.00	-	-	-
Female	0.46	1.00	-	-	-
C3 Fear of public criticism (total)	0.39	0.35	1.00	-	-
Male	0.39	0.35	1.00	-	-
Female	0.38	0.33	1.00	-	-
C4 Fear of a family member getting infected (total)	0.37	0.28	0.39	1.00	-
Male	0.37	0.28	0.40	1.00	-
Female	0.37	0.27	0.38	1.00	-
C5 Fear of economic loss due to infection (total)	0.31	0.29	0.34	0.42	1.00
Male	0.32	0.29	0.36	0.43	1.00
Female	0.29	0.28	0.32	0.40	1.00

**Table 3.** Results of the multivariable linear regression analysis

Variables	Disruption in daily activities		
	Adjusted-β^1^	SE	p-value
Fear of infection			
No	Reference		
Yes	-3.37	0.17	<0.001
Fear of dying from infection			
No	Reference		
Yes	-0.33	0.15	0.030
Fear of public criticism			
No	Reference		
Yes	-1.63	0.17	<0.001
Fear of a family member getting infected			
No	Reference		
Yes	-1.03	0.21	<0.001
Fear of economic loss due to infection			
No	Reference		
Yes	-3.52	0.18	<0.001
Sex			
Male	Reference		
Female	-3.02	0.15	<0.001
Age (yr)			
19-29	Reference		
30-39	-3.79	0.31	<0.001
40-49	-6.03	0.32	<0.001
50-59	-4.28	0.31	<0.001
60-69	-2.94	0.29	<0.001
≥70	-3.07	0.25	<0.001
Education			
No diploma	Reference		
Elementary school	-3.07	0.36	<0.001
Middle school	-4.35	0.39	<0.001
High school	-4.76	0.37	<0.001
College or above	-5.33	0.39	<0.001
Income level			
Low	Reference		
Low-middle	-0.11	0.23	0.648
Middle-high	0.01	0.24	0.955
High	0.28	0.25	0.268
Job classification			
Professional or administrative work	Reference		
Office work	1.28	0.25	<0.001
Sales and service	-1.57	0.26	<0.001
Agriculture and fishery	1.47	0.33	<0.001
Blue-collar work or simple labor	0.47	0.25	0.060
Unemployed	-2.55	0.23	<0.001
Household composition			
One generation	Reference		
Two generations	-0.37	0.16	0.023
Three generations	-0.48	0.27	0.079
Area of residence			
Rural	Reference		
Urban	-2.13	0.15	<0.001
Drinking at least once per month			
No	Reference		
Yes	-0.75	0.14	<0.001
Smoking			
No	Reference		
Yes	-0.52	0.20	0.008
Depressive symptoms (PHQ-9 ≥10)			
No	Reference		
Yes	-3.15	0.48	0.004
Perceived stress			
No	Reference		
Yes	-4.49	0.16	<0.001
Subjective health status			
Poor	Reference		
Fair	1.04	0.14	<0.001

SE, standard error; PHQ-9, Patient Health Questionnaire-9.

1 Adjusted for sex, age, education, income level, job classification, household composition, area of residence, drinking at least once per month, smoking, depressive symptoms, perceived stress, and subjective health status.

**Table 4.** Results of the multivariable linear regression analysis

Variables	Disruption in daily activities		
	Adjusted-β^1^	SE	p-value
No. of COVID-19-related fears experienced			
0	Reference		
1	-2.71	0.37	<0.001
2	-5.31	0.33	<0.001
3	-7.41	0.31	<0.001
4	-9.78	0.30	<0.001
5	-10.34	0.30	<0.001

SE, standard error; COVID-19, coronavirus disease 2019.

1 Adjusted for sex, age, education, income level, job classification, household composition, area of residence, drinking at least once per month, smoking, depressive symptoms, perceived stress, and subjective health status.

**Table 5.** Results of the subgroup analysis by income

Income level	Disruption in daily activities		
	Adjusted-β^1^	SE	p-value
No. of COVID-19-related fears experienced			
Low			
0	Reference		
1	-4.10	0.98	<0.001
2	-6.45	0.86	<0.001
3	-8.97	0.79	<0.001
4	-11.98	0.76	<0.001
5	-13.13	0.71	<0.001
Middle-low			
0	Reference		
1	-3.63	0.89	<0.001
2	-5.48	0.78	<0.001
3	-7.01	0.73	<0.001
4	-10.11	0.70	<0.001
5	-10.75	0.69	<0.001
Middle-high			
0	Reference		
1	-2.51	0.72	0.001
2	-5.89	0.64	<0.001
3	-8.11	0.59	<0.001
4	-10.29	0.57	<0.001
5	-10.52	0.56	<0.001
High			
0	Reference		
1	-1.82	0.59	0.002
2	-4.27	0.53	<0.001
3	-6.42	0.50	<0.001
4	-8.38	0.49	<0.001
5	-8.68	0.49	<0.001

SE, standard error; COVID-19, coronavirus disease 2019.

1 Adjusted for sex, age, education, job classification, household composition, area of residence, drinking at least once per month, smoking, depressive symptoms, perceived stress, and subjective health status.
